# Dyslipidemia in Anorexia Nervosa Is Associated with Decreased Plasma Tauroursodeoxycholic Acid and a Specific Fatty Acid Pattern

**DOI:** 10.3390/nu17142347

**Published:** 2025-07-17

**Authors:** Aleš Žák, Marek Vecka, Peter Szitanyi, Marcela Floriánková, Barbora Staňková, Petra Uhlíková, Veronika Dostálová, Michal Burda

**Affiliations:** 1 4th Department of Medicine, 1st Faculty of Medicine, Charles University in Prague and the General University Hospital in Prague, 128 08 Prague, Czech Republic; azak@vfn.cz (A.Ž.); marcela.floriankova@vfn.cz (M.F.); barsta@atlas.cz (B.S.); 2 Institute of Clinical Chemistry and Laboratory Diagnostics, 1st Faculty of Medicine, Charles University in Prague and the General University Hospital in Prague, 128 08 Prague, Czech Republic; 3 Department of Pediatrics and Inherited Metabolic Disorders, 1st Faculty of Medicine, Charles University in Prague and the General University Hospital in Prague, 128 08 Prague, Czech Republic; szitanyi@yahoo.com (P.S.); petra.uhlikova@vfn.cz (P.U.); 4 Department of Psychiatry, 1st Faculty of Medicine, Charles University in Prague and the General University Hospital in Prague, 128 08 Prague, Czech Republic; veronika.dostalova2@vfn.cz; 5 Institute for Research and Applications of Fuzzy Modeling, CE IT4Innovations, University of Ostrava, 701 03 Ostrava, Czech Republic; michal.burda@osu.cz

**Keywords:** anorexia nervosa, lipids and lipoproteins, fatty acid pattern, plasma bile acid composition, multiple linear regression analysis, long-chain polyunsaturated fatty acids of *n*-6 family

## Abstract

Background: Dyslipidemia and distorted fatty acid (FA) metabolism are frequent biochemical abnormalities associated with anorexia nervosa (AN). Gut microbiota is supposed to play an important role in the etiopathogenesis of AN. Apart from the digestive function of bile acids (BAs), these compounds have multiple metabolic functions due to the activation of specific receptors. Objective/aims: The aims of the study were to investigate biochemical measures, including plasma lipids (lipoproteins, respectively), fatty acid (FA) patterns, and the profile of plasma Bas, in AN patients and healthy controls (CON). Methods: Plasma phospholipid FA and BAs profiles were analyzed in 39 women with a restrictive type of AN (AN-R; median age 17 years) and in 35 CON women (median age 20 years). Results: Compared to CON, AN had an increased concentration of HDL-C, increased content of palmitic acid, and decreased proportion of linoleic acid. Moreover, AN had a drop in the level of the sum of PUFA*n*-6 and increased delta 9 desaturase activity for stearic acid. In AN, we found decreased levels of plasma tauroursodeoxycholic acid (TUDCA). In AN, concentrations of 22:5*n*-6, 16:0, 20:3*n*-6 and fat mass index were predic-tors of HDL-C levels (R^2^ = 0.43). Conclusions: Patients with AN-R had an increased concentration of HDL-C, decreased levels of total PUFA *n*-6, and increased activity of D9D for stearic acid. Furthermore, AN exerted decreased levels of TUDCA. Therefore, a decreased level of TUDCA could potentially serve as a marker of AN.

## 1. Introduction

Anorexia nervosa (AN) is a serious psychiatric disorder with a peak onset in both men and women between 15 and 19 years, which has the highest mortality and relapse rate of all psychiatric illnesses. AN is characterized by persistent, pathological weight and shape concerns, leading to a restricted oral intake and, consequently, low body weight, with a reduction in fat mass and fat-free mass at the expense of adipose tissue [1,2,3].

The etiology of AN remains unclear and is supposed to be complex and multifactorial, with substantial environmental and genetic influences. The main risk factors predisposing individuals to the development of AN include environmental and social aspects, biological influences, psychological traits, and genetic factors, with the heritability of AN ranging from 20 to 58% [4,5,6].

Energy deprivation and malnutrition have a major impact on several organs and systems in patients with AN. Patients with AN present multiple psychiatric and somatic comorbidities/complications related to prolonged starvation and altered eating behaviors. The most common psychiatric comorbidities include mood and anxiety disorders, obsessive-compulsive disorder, personality traits (anxiety, depression, perfectionism, and low self-esteem), substance use disorders, and neurodevelopmental disorders (autism spectrum and attention deficit hyperactivity disorder) [7]. Furthermore, AN is associated with several somatic comorbidities.

The phenotype of AN depends on many factors, such as the age at AN onset, duration, and severity of illness [3,7,8]. AN is associated with several somatic comorbidities, the most serious of which are cardiac complications (Supplementary Table S1).

Dyslipidemia and impaired fatty acid (FA) metabolism are the common biochemical abnormalities found in AN. Lipid (lipoprotein, respectively) alterations in AN have been known since the 1960s [9,10]. The results of studies analyzing lipid (lipoprotein, respectively) concentrations in AN seem to be inconsistent. Several studies have described hypercholesterolemia in 20 to 50% of patients with AN [11,12,13], whilst other studies found normal or reduced plasma lipid concentrations. On the other hand, Crisp et al. [10] found nearly normal levels of plasma total cholesterol (TC), with only two persons out of twelve who were hypercholesterolemic. Other authors [14] have described only elevated levels of HDL-C in AN. However, in normolipidemic patients with AN, changes in the lipoprotein spectrum toward more atherogenic subclasses have been described [15]. A large-scale meta-analysis described the effect of AN on plasmatic TC, LDL-cholesterol (LDL-C), HDL-cholesterol (HDL-C), and triacylglycerols (TAG). Patients with acute AN had, compared to healthy controls, on average, increased levels of TC by 0.60 mmol/L, LDL-C by 0.32, HDL-C by 0.09, and TAG by 0.09 mmol/L [16].

The pathophysiological mechanisms underlying hypercholesterolemia in anorexia nervosa are not yet fully understood; increased cholesterol synthesis seems unlikely [17]. Other studies have suggested increased absorption of exogenous cholesterol [18,19]. Several studies have described a disturbed hormonal milieu that could lead to secondary dyslipidemia (reviewed in Refs. [16,17,20]).

Dysregulated plasma fatty acid (FA) patterns have been found in patients with eating disorders (EDs) such as obesity and AN in several studies [21,22,23,24]. Apart from inconsistent changes in concentrations of saturated (SFAs) and monounsaturated FAs of the n-9 family (MFA n-9), the most consistent changes in the FA profiles in AN patients include alterations in polyunsaturated FAs (PUFAs) [25].

Among the biological factors involved in the etiopathogenesis of AN, the gut microbiota plays a significant role. In recent years, the importance of the gut microbiota in the pathogenesis of eating disorders (ED) such as AN and bulimia nervosa has been intensively studied [7,26,27,28]. The composition of the gut microbiota is highly variable between individuals and depends on many endogenous and exogenous factors. The most important factor that influences the size and composition of gut microbiota is the diet [29,30].

It is generally accepted that the gut microbiota plays a crucial role in the gut microbiota–brain axis. Gut dysbiosis (disturbed size and composition of the gut microbiota) influences communication between the gut/pancreas, brain, and adipose tissue. The gut microbiota affects the synthesis of many compounds/substances, such as hormones and short-chain fatty acids (SCFAs), with an impact on intestinal and blood–brain barrier permeability, subclinical inflammation of the intestine and central nervous system (CNS), the expression of hormones and neurotransmitters affecting appetite and satiety [28]. 

Furthermore, the gut microbiota significantly affects the size and composition of the bile acid (BA) pool. BAs have been recognized as signaling molecules with many pleiotropic physiological functions. BAs act as metabolic integrators of lipid, glucose, and energy metabolism. BAs also control gastrointestinal motility, inflammation, size, and configuration/growth of the gut microbiome.

The BA pool in the human body is maintained by efficient enterohepatic circulation, preserving up to 95% of conjugated BA. Absorbed BA enter the portal bloodstream and are rapidly taken up by hepatocytes and resecreted into bile. A small fraction of BA escapes enterohepatic recirculation and spills into the systemic circulation, which allows BA signaling to occur in other organs and tissues. Only a small amount (~5%) of BA enters the colon, where the gut microbiota can convert primary BA into more hydrophobic secondary BAs—deoxycholic acid (DCA) and lithocholic acid (LCA). These secondary BAs can be passively reabsorbed from the colon. Primary BAs are further metabolized in the intestine by the gut microbiota in processes that involve the action of three groups of bacterial enzymes: bile salt hydrolases, hydroxysteroid dehydrogenases that oxidize and epimerize hydroxy groups of the BA molecule, yielding the secondary/tertiary BAs—ursodeoxycholic acid (UDCA), iso-DCA, and iso-LCA. The third reaction is the 7α-dehydroxylation of unconjugated BA in the colon. In the intestine, conjugated primary BA [cholic acid (CA), chenodeoxycholic acid (CDCA), respectively] can be deconjugated and 7α-dehydroxylated by the gut microbiota to form secondary (DCA, LCA, UDCA, respectively). The gut microbiota controls BA diversity and vice versa; the size of the BA pool and its composition mediate gut microbiome patterns [31,32,33]. However, until now, changes in the size and composition of plasma BA have not been systematically studied in AN.

TUDCA exerts many beneficial functions in human metabolism: (a) TUDCA stabilizes the endoplasmic reticulum thus inhibiting endoplasmic reticulum stress [34] that induces leptin resistance [35] and activates several inflammatory pathways [36]; (b) stabilizes mitochondrial membranes, probably acting as molecular chaperone by blocking the activation of unfolded protein response and consequent apoptosis [37]; (c) activates TGR5 receptors which results in the attenuation of the production of proinflammatory cytokines, also during activation of microglia and astrocytes [38]. Neuroinflammatory processes are known to be present in the hypothalamus in AN [39]. In obesity models, TUDCA restored hypothalamic sensitivity to leptin. In AN, hypothalamic leptin signalization also changes [40].

In our previous study, changes in plasma lipids, fatty acid pattern, and non-cholesterol sterols (lathosterol, β-sitosterol, campesterol) were found in AN [18]. The aims of the study were to investigate clinical features, biochemical measures that include plasma lipids (lipoproteins, respectively), FA patterns, and the profile of plasma BA in AN patients and healthy controls (CON). Furthermore, using multiple linear regression analysis, concentrations of plasma lipids/lipoproteins (as dependent variables) and levels of individual FAs (as independent variables) were studied in both groups (i.e., AN and CON).

## 2. Materials and Methods

### 2.1. Study Design and Participants

This case-control study was carried out at the Department of Pediatrics and Inherited Metabolic Disorders and the 4th Department of Medicine. Thirty-nine women with a restrictive type of anorexia nervosa (AN-R), recruited from the consecutive outpatients of the Department of Pediatrics and Inherited Metabolic Disorders from September 2018 to August 2022, and thirty-five sex and age-matched healthy controls (CON) were enrolled in the study. All women studied were of Caucasian descent. All patients with AN-R met the Diagnostic and Statistical Manual-V [1] criteria of this illness, and diagnosis was confirmed by two independent pediatric and adolescent psychiatrists/psychologists.

The study protocol was approved by the Ethics Committee of the General University Hospital in Prague (decision no. 611/18 S; 2018) and performed in accordance with the principles of the Declaration of Helsinki. Written informed consent was obtained from all participants. The medical examination was performed during the acute stage of the disease. The patients did not suffer from any somatic disorder. A bulimic type of anorexia was excluded. Other exclusion criteria were chronic somatic diseases or other mental/neurodevelopmental disorders (a primary disease related to eating disorders), hereditary disorders (first-degree, such as familial hypercholesterolemia), pharmacotherapy, hormonal therapy, pregnancy, contraception, dietary supplements, and smoking. The patients and controls did not use drugs known to affect lipid and lipoprotein metabolism, vitamins, antioxidants, and supplements enriched with *n*-3 and/or *n*-6 PUFA. The control group consisted of eumenorrheic and generally healthy girls who agreed to participate in the study. Patients attending urgent or non-routine treatment were excluded from the control group. No control subject reported eating disorders in the past. Other exclusion criteria in the CON group related to general health were chronic somatic diseases, mental or neurodevelopmental disorders, hereditary disorders, such as familial dyslipidemia (in first-degree relatives), pregnancy or breastfeeding, pharmacotherapy, dietary supplements, or contraception. Neither a family history of premature coronary heart disease nor familial dyslipidemia was detected.

### 2.2. Blood Sampling, Diet Evaluation, and Anthropometry

Blood samples were taken after 12 h of fasting. Routine biochemical and hematological analyses were performed immediately, and samples for special analyses were stored at −80 °C until use. Basic clinical, nutritional, and anthropometric data, including assessment of body fat, were examined using standard methods, as described previously [18,41].

### 2.3. Indirect Calorimetry

Resting energy expenditure (REE) and respiratory quotient were analyzed with a computerized, open-circuit indirect calorimetry system that measured resting oxygen uptake and resting carbon dioxide production using a ventilated canopy (Omnia 1.5—Quark RMR, Cosmed, Srl, Italy, Rome).

### 2.4. Laboratory Measurements

Total cholesterol (TC) was analyzed in serum using a commercially purchased enzymatic-colorimetric test (CHOD-PAP) (Biola-test cholesterol 2500 kit; Pliva-Lachema, Czech Republic). Serum TAGs were measured using a standard enzymatic-colorimetric kit (GPO-PAP) (Pliva-Lachema, Czech Republic). HDL-C concentration was measured in the supernatant of serum samples after selective precipitation of LDL-C using a BIO-LA-TEST HDL-Cholesterol kit (Pliva-Lachema, Czech Republic) on a Cobas Mira analyzer (Roche, Switzerland). The levels of non-esterified fatty acids (NEFAs) were determined using an enzymatic-colorimetric method (Randox Laboratories Ltd., Crumlin, UK). Apolipoprotein B concentration was analyzed nephelometrically using a standard kit (Beckman Coulter Inc., Brea, CA, USA) on an Image analyzer. Insulin concentrations were measured using diagnostic sets and a modular analyzer (Roche Diagnostic, Indianapolis, IN, USA) by electrochemiluminescence.

The fatty acid profiles in the main plasma lipid classes (phospholipids, triacylglycerols, and cholesteryl-esters) were examined by analytical procedures described previously [32]. The homeostasis model assessment method, HOMA-IR, was used as an index of insulin resistance [33]. Desaturase activities were estimated using FA product/precursor ratios [42,43]. The ratio of palmitoleic to linoleic acid (16:1*n*-7/18:2*n*-6) greater than 0.086 was used as a surrogate marker of *n*-6 essential fatty acid deficiency (EFAD) [44]. The plasma BA spectrum was measured by the LC-MS/MS method [45].

### 2.5. Statistical Analyses

All statistical analyses were performed using the R statistical software version 4.1.3. or with STATISTICA^®^ software version 12 for Windows [46]. Categorical data were summarized by absolute and relative frequencies. Continuous data are expressed as median and interquartile range (IQR, 25th–75th percentile). The normality of the distribution was tested using the Shapiro–Wilk test. Comparisons between groups were carried out using the two-sample Mann–Whitney test. The *p*-values for continuous and categorical data were adjusted for multiple comparisons using Holm’s correction.

Stepwise multivariate linear regression was used to assess the relationship between plasma lipids (lipoproteins, respectively), such as dependent variables, and plasma phospholipid FA, body mass index (BMI), fat mass index (FMI), glucose, and insulin, such as independent variables. To avoid collinearity, these relationships were observed using two models. In the first model, the concentrations of FA and the values of BMI and FMI were included as independent variables; in the second model, concentrations of FA, insulin, and glucose were used as independent variables. A stepwise multivariate linear regression analysis was performed to determine the coefficients of determination (R^2^) using FA concentrations, anthropometric measures (BMI and FMI), and biochemical parameters (glucose and insulin).

We performed post hoc power analyses to better quantify the sensitivity of the statistical tests. For comparisons based on the largest available subsample (*n* = 39 + 35), the analyses indicate a power of 0.89 to detect large effect sizes (Cohen’s d ≈ 0.8, α = 0.05, two-tailed). However, for smaller subsamples (e.g., *n* = 21 + 13), the power to detect large effects drops to 0.56. This illustrates that while some parts of the study were sufficiently powered to detect large effects, other parts were underpowered, particularly to detect moderate or small effects. We have clarified in the text that due to these limitations, the findings should be interpreted with caution and viewed as exploratory. The results may serve as a basis for generating hypotheses to be tested in future studies with larger and more balanced samples.

## 3. Results

### 3.1. Basic Clinical, Anthropometric, and Biochemical Characteristics

The basic clinical, anthropometric, and biochemical characteristics of the study groups (i.e., AN, CON) are shown in Table 1. The anthropometric parameters of patients with AN are coherent with the marantic type of malnutrition (simple starvation, respectively). As expected, the patients with AN had, in comparison with the healthy CON group, decreased BMI (by 21%), fat mass (by 51%), lean body mass (by 13%), and fat mass index (by 45%). These data show that weight loss is linked with adipose tissue reduction. The AN group had decreased REE (by 36%), levels of triiodothyronine as well as thyroxine (by 45%, and by 21%, respectively). Biochemical characteristics are completed by a reduced insulin concentration (by 55%) and a decrease in the HOMA-IR index (by 62%). In the AN group, we found only an increase in the concentration of HDL-C fraction (by 22%). Although the number of patients with high concentrations of HDL-C (>2.07 mmol/L), considered as dysfunctional, was higher in the AN group (4 of them met the criteria), we did not prove a significant difference in the distribution (chi-square test with Yates‘ correction; *p* = 0.422). Dysfunctional HDL is not believed to have protective effects in relation to cardiovascular disease [47,48].

The analyses of CRP concentrations did not reveal differences between the studied groups. However, the molar ratio of TUDCA exhibited a negative correlation with the CRP values in the AN group (r = −0.58; *p* = 0.05, Pearson correlation coefficient).

The composition of the diets of both studied groups is presented in Supplementary Table S5. The AN group had lower energy and fat intake, as documented by lower cholesterol and saturated fat content in the diet.

### 3.2. Fatty Acid Profiles

The plasma phospholipid FA profile is shown in Table 2. In the group with AN, we found, in comparison with healthy controls, a statistically significant increased concentration of palmitic acid (PA, 16:0) by 10%, and a decreased level of linoleic acid (LA; 18:2*n*-6). Anorectic patients had decreased concentrations of the sum of all polyunsaturated fatty acids (ΣPUFA *n*-6) and an increased index of D9D 18 (delta 9 desaturase for stearic acid) (Table 3). Neither patients in the AN group nor subjects in the CON group showed biochemical signs of essential fatty acid deficiency using surrogate markers (Table 3).

### 3.3. Bile Acid Composition

When comparing the profile of plasma bile acids, we found reduced concentrations of tauroursodeoxycholic acid (TUDCA) in the AN group, both when evaluating absolute concentrations [0.003 (0.002–0.006) vs. 0.010 (0.006–0.021); μmol/L; median (25th–75th percentile), *p* < 0.05)], and when comparing their proportion [0.140 (0.068–0.251) vs. 0.397 (0.295–0.493); mol%; median (25th–75th percentile) *p* < 0.05] (Supplementary Tables S2 and S3). Although the data were available only for a subset of the studied groups (21 participants in the AN group and 13 individuals in the CON group), we did not observe a selection bias between those with analyzed BA concentrations and those without the BA measurements.

### 3.4. Regression Analysis

The results of multivariate linear regression analysis between plasma lipids (lipoproteins, respectively, that is, TC, LDL-C, HDL-C, TAG, apo B, and LDL-C/HDL-C) as dependent variables and plasma phospholipid FA, BMI, FMI, glucose, and insulin as independent variables in the AN patients and CON group are given in Table 4/Supplementary Table S4. In the AN group, a moderate correlation (R^2^ = 0.43) was found between FA (22:5*n*-6, 16:0, 20:3*n*-6), FMI, and HDL-C, as well as between FA (20:0, 16:1*n*-9), insulin, and TAG (R^2^ = 0.45). The coefficients of determination between TC and LDL-C, and plasma FAs were mild (R^2^ = 0.28–0.33), while the independent variables were 22:4*n*-6 and 18:1*n*-9 for TC and LDL-C.

In the CON group, a weak correlation was found between HDL-C and the 20:0 level (R^2^ = 0.15). The moderate correlation between the TAG level and plasma phospholipid FA was found in both models. TAG levels were dependent on levels of 22:5*n*-6 and 20:5*n*-3 (R^2^ = 0.37). Similarly, a moderate (strong resp.) correlation between TC and LDL-C (R^2^ = 0.46, 0.64, resp.) and 16:0, and the sum of PUFA n-6 was found. Apo B levels were dependent on four FAs (16:0, 18:2*n*-6, 20:4*n*-6, and 20:0) and BMI, with a strong coefficient of determination (R^2^ = 0.61).

As it follows from the regression analysis, an increase in HDL-C concentration is associated with a decrease in DPA-6 (22:5*n*-6; 4,7,10,13,16-docosapentaenoic acid) and DHGLA (dihomo-γ-linolenic acid, 20:3*n*-6). In contrast, an increase in TC (LDL-C, respectively) is associated with an increase in adrenic acid (22:4*n*-6; 7,10,13,16-docosatetraenoic acid) and a decrease in oleic acid (18:1*n*-9).

## 4. Discussion

The main results of our study are the association of HDL-C concentrations with changes in the concentrations of specific FA and the finding of reduced TUDCA concentrations in AN. The clinical and biochemical data of our study are consistent with the published results of other authors [3,16,49,50]. In agreement with the literature [14,16], we demonstrated only a significant increase in HDL-C concentration in AN. In our previous study, we demonstrated an increase in TC, TAG, and HDL-C, as well as an elevation in NEFA levels in AN [18]. This difference may be due to a general change in the dietary habits of the Czech population in recent years [51].

In the group with AN, we found, compared to healthy controls, a statistically increased content of palmitic acid (PA, 16:0) by 10%, and a decreased level of linoleic acid (LA; 18:2*n*-6). Anorectic patients had decreased concentrations of the sum of all polyunsaturated fatty acids (ΣPUFA *n*-6) as well as increased index of D9D 18 (delta 9 desaturase for stearic acid. These results are consistent with the results of our previous study, i.e., an increase in 16:0, 18:1*n*-9, SFA, and MFA, and a decrease in *n*-6 PUFA and 18:2*n*-6 [18].

Changes in FA metabolism in ED (especially in AN) have been studied by several authors. Nguyen et al. [52] found an increased concentration of lauric acid (12:0), eicosapentaenoic acid (EPA, 20:5*n*-3), DPA-3 (22:5*n*-3), and ALA (α-linolenic acid, 18:3*n*-3) in AN compared to healthy controls, while food intake shifted the pattern of FA toward a less pathological FA profile. French authors studied the composition of FA in plasma phospholipids and erythrocyte membranes. They did not find EFAD, a decrease in LA, or ALA, respectively, and no increase in 20:3*n*-9. However, they observed decreased levels of LC-PUFA, mainly those in the *n*-3 family [53]. A meta-analysis investigating the composition of PUFAs in ED (mainly in AN, 6 of 7 studies) found, compared to controls, higher plasma levels of ALA, EPA, stearidonic acid (18:4*n*-3), Osbond acid (DPA*n*-6; 22:5*n*-6), palmitoleic acid (POA, 16:1*n*-7), oleic acid (18:1*n*-9), and total PUFA *n*-3 fatty acids, together with lower levels of total n-6 fatty acids and a ratio of PUFA*n*-6/PUFA*n*-3. Eating disorders were associated with significantly higher levels of palmitoleic acid and oleic acid in the red blood cell membrane and lower levels of adrenic acid (22:4*n*-6), arachidonic acid (20:4*n*-6), and total n-6 fatty acids [24]. Japanese authors in a case-control study found in AN-R, compared to controls, increased levels of 18:3*n*-3, 20:0, 20:1*n*-9, 20:5*n*-3, 22:0, and 22:1*n*-9 [23].

Differences in the composition of FAs when comparing our work with that of other authors may be partly due to methodological aspects. In our study, we express the individual FA in mol%, just like some other authors [24]; other authors express the concentrations of FAs in absolute units (μmol/L) [23,25,52]. We did not find indirect signs of EFAD [LA/POA ratio] or Mead’s acid content in the AN group.

The relationship between the amount of fat in the diet and its qualitative composition and human health, especially the occurrence of cardiovascular diseases, has been known since the late 1950s [54]. In subsequent years, research focused on the effects of individual saturated FAs, trans MFA, cis MFA, *n*-6 and *n*-3 PUFA on plasma lipids, and lipoprotein (including lipoprotein [a]), and enzymes of lipoprotein metabolism (reviewed in Refs. [55,56]). HDL-C concentrations in AN were positively associated with PA and negatively associated with DPA-6 and DHGLA. This means that a decrease in DPA-6 and DHGLA was reflected in AN by an increase in HDL-C concentration, and an increase in PA similarly leads to an increase in HDL-C. The lack of LC PUFA n-6 is probably causally related to the increase in HDL-C in AN. DPA-6 is an indicator of DHA (docosahexaenoic acid, 22:6*n*-3) deficiency and is related to several other disease states [24,57,58,59].

In the study by Muralidharan et al., a relationship was demonstrated between the composition of LC-PUFA n-6 in cell membranes, the size and function of HDL, and peripheral inflammation (levels of interleukin-6 and -8, respectively) [60]. Furthermore, patients with phenylketonuria revealed changes in plasma *n*-6 PUFA [higher LA, adrenic acid (AdA), and DPA-6] [61]. Women with gestational diabetes had lower concentrations of DHGLA, DHA, arachidonic acid (AA), AdA, DPA-6, and the AdA/DPA-6 ratio, which is a biochemical marker of DHA insufficiency [62]. On the other hand, a study by Dutch authors did not demonstrate a relationship between AA, AdA, DPA-6, EPA, and DHA acids and cognitive performance in non-pregnant women [63].

In a large meta-analysis of 379 patients with ED, higher plasma concentrations of ALA, EPA, 18:4*n*-3, DPA-6, POA, 18:1*n*-9 (oleic acid), and ΣPUFA*n*-6 and a higher ratio of *n*-6 PUFA to *n*-3 PUFA were found, as well as lower concentrations of AdA, AA, and the total *n*-6 PUFA [24].

Changes in liver and plasma phospholipids were demonstrated in obese patients. Obese patients showed lower levels of AA, DPA-3, DHA, total LC-PUFA, and total *n*-3 LC-PUFA and higher levels of DPA*n*-6, and higher ratios of *n*-6/*n*-3 LC-PUFA. Obese patients with nonalcoholic fatty liver disease showed marked alterations in the PUFA patterns in the liver. These changes are significantly correlated with those found in erythrocytes [64]. Zec et al. (2019) described the association of LC-PUFA *n*-6 with blood pressure and hypertension in black South African adults [58]. These authors found a negative association of DPA-6 with diastolic blood pressure [58]. The levels of DPA-6 (AdA/DPA-6 ratio) are considered a biochemical marker of the functional DHA status [59].

A surprising finding was the positive association of arachidic acid (20:0) with HDL-C levels in the CON group and the negative association of 20:0 with TAG in the AN group, and its positive association of 20:0 with apo B concentration in the CON group. These associations reached only low-to-moderate strength. Arachidic acid is a very-long-chain saturated fatty acid (VLCSFA). The concentrations of VLCSFA show negative associations with the risk of CV disease and are also a negative indicator of de novo lipogenesis. Increased VLCSFA concentrations are a biochemical marker of reduced lipogenesis. They appeared to be directly involved in reduced lipogenesis [65,66].

Changes in the gut microbiota, the synonym “dysbiosis”, are characterized by low microbial diversity, the presence of pathobionts, and the lack of beneficial bacteria. Dysbiosis has been described in a growing number of diseases, such as in patients with inflammatory bowel disease, irritable bowel syndrome, asthma, obesity, and various neuropsychiatric disorders, including AN [67,68]. The decreased plasma TUDCA concentration, found in our study, is likely a surrogate marker of intestinal dysbiosis. Furthermore, decreased plasma TUDCA concentrations may be involved in the etiopathogenesis of AN through at least two other mechanisms. As we did not analyze SCFA concentrations or gut microbiota composition, these phenomena cannot be supported by our data. The gut microbiota, in addition to the gastrointestinal tract and CNS, are involved in the synthesis of several compounds with orexigenic (appetite-stimulating) and anorexigenic (appetite-suppressing) effects. Balance and interactions between the anorexigenic α–melanocyte-stimulating hormone, cholecystokinin, peptide YY, and orexigenic peptides (ghrelins) originating from the gastrointestinal tract appear to play an important role in the regulation of food intake by affecting hypothalamic energetic homeostasis (satiety, resp.) regulating centers. The imbalance influences feeding behavior with subsequent weight loss or weight regain in AN patients [69].

Experimental studies have shown that plasma concentrations of BA correlate with their levels in the brain [70]. Therefore, we can assume that TUDCA concentrations in the central nervous system might be reduced in patients with AN. In recent years, TUDCA has shown important anti-apoptotic and neuroprotective activities, with numerous experimental and clinical evidence suggesting its possible therapeutic use as a disease-modifier in neurodegenerative diseases. Experimental evidence on the mechanisms underlying TUDCA’s neuroprotective action derives from animal models of Alzheimer’s disease, Parkinson’s disease, Huntington’s disease, amyotrophic lateral sclerosis, and cerebral ischemia. Preclinical studies indicate that TUDCA exerts its effects not only by regulating and inhibiting the apoptotic cascade but also by reducing oxidative stress, protecting mitochondria, producing an anti-neuroinflammatory action, and acting as a chemical chaperone to maintain the stability and correct folding of proteins [71]. Therefore, the lack of TUDCA in the CNS could potentiate and/or exacerbate low-grade inflammation and neurodegenerative changes in AN.

Another mechanism by which changes in the spectrum of BA can be applied in AN is the anorexigenic effects of BA, which are mediated by receptor TGR5 [72,73]. However, individual BAs and free, tauro-, and glycoconjugates show different activity towards TGR5 and TUDCA, as the most hydrophilic BA has the lowest affinity for TGR5 [74,75]. Therefore, it is unlikely that a decrease in TUDCA contributes to the anorexigenic effect in AN.

### Strengths and Limitations of the Study

This study had several strengths. To our knowledge, this study is the first to report decreased plasma concentrations of TUDCA in AN. Second, the experimental group consists exclusively of subjects with a restrictive type of AN, without psychiatric treatment and any nutritional support. Third, we present an innovative statistical approach evaluating the dependence of plasma lipid (lipoprotein, respectively) concentrations on FA levels in plasma phospholipids.

This study has some limitations. First, this is a retrospective cross-sectional study with a relatively small number of individuals in both groups. Second, not all subjects in both groups had analyses of BA concentrations, and third, there was a lack of intestinal microbiota and SCFA analyses.

## 5. Conclusions

Patients with AN-R had an increased concentration of HDL-C, decreased levels of the total PUFA *n*-6, and increased activity of D9D for stearic acid. Furthermore, AN exerts decreased levels of TUDCA. The HDL-C level was determined by levels of 22:5*n*-6, 20:3*n*-6, 16:0, and FMI. TC, LDL-C, and the LDL-C/HDL-C ratios were influenced by 22:4*n*-6 and 18:1*n*-9. In conclusion, dyslipidemia (increased HDL-C) is associated with a specific FA pattern.

The unexpected finding of decreased TUDCA levels, probably due to changes in the gut microbiota, could potentially serve as a marker of AN. The considerations regarding the possibility of correcting the decrease in TUDCA by its substitution are still speculative and will be the subject of further research.

## Figures and Tables

**Table 1:** Basic clinical and biochemical characteristics of the studied groups.

Parameter	AN (*n* = 39)	CON (*n* = 35)
Age (years)	17 (14–21) ^1^	20 (16–22)
BMI (kg·m^−2^)	16.4 (13.6–18.2) ***	20.8 (19.8–22.4)
Fat mass (kg)	7.6 (4.2–10.6) ***	15.5 (12.4–18.5)
Fat mass index (kg·m^−2^)	3.1 (1.5–4.0) ***	5.6 (4.8–6.5)
Lean body mass (kg)	36.3 (30.2–41.1) *	41.8 (39.1–43.6)
LBMI (kg·m^−2^)	13.3 (11.7–15.0) **	15.4 (14.4–16.3)
REE (kcal)	998 (866–1309) **	1569 (1503–1645)
NEFA (mmol/L)	0.410 (0.155–0.780)	0.420 (0.322–0.532)
T3I (nmol/L)	3.350 (2.775–3.855) ***	6.100 (5.900–6.500)
T4I (nmol/L)	12.300 (11.750–13.225) **	15.500 (14.200–16.100)
TSH (mIU/L)	1.928 (1.375–2.280)	2.670 (1.943–3.821)
TC (mmol/L)	4.76 (4.23–5.59)	4.18 (3.95–4.88)
TAG (mmol/L)	0.92 (0.80–1.24)	0.73 (0.61–1.03)
Apo B (g/L)	0.74 (0.60–0.85)	0.72 (0.64–0.77)
HDL-C (mmol/L)	1.56 (1.40–1.85) ***	1.28 (1.07–1.42)
LDL-C (mmol/L)	2.62 (2.18–3.31)	2.61 (2.24–3.20)
Non-HDL-C	3.16 (2.390– 3.96)	3.07 (2.69–3.54)
LDL/HDL (ratio)	1.74 (1.24–2.25)	2.14 (1.72–2.84)
Glucose (mmol/L)	4.6 (4.1–5.0)	4.7 (4.5–4.9)
Insulin (mU/L)	5.12 (3.57–6.36) **	11.36 (7.84–16.30)
HOMA-IR (ratio)	0.98 (0.59–1.56) *	2.57 (1.57–3.26)
CRP (mg/L)	2.10 (0.54–4.38)	2.30 (0.50–4.38)

^1^ Data are given as median (25th–75th percentile); statistical analysis: Mann–Whitney test: * *p* < 0.05, ** *p* < 0.01, *** *p* < 0.001; after adjustment for multiple comparisons using Holm’s corrections. Abbreviations: AN—anorexia nervosa; BMI—body mass index; CON—control group; CRP—C-reactive protein; TC—total cholesterol; TAG—triacylglycerols; LDL—low density lipoproteins; HDL—high density lipoproteins; Apo—apolipoprotein; HOMA-IR—homeostasis model assessment for insulin resistance (f-insulin (μU/mL) × f-glucose (mmol/L)/22.5); REE—resting energy expenditure; T3I—triiodothyronine; T4I—thyroxine; TSH—thyroid stimulating hormone; NEFA—non-esterified fatty acids; LBMI—lean body mass index; FMI—fat mass index.

**Table 2:** Plasma phospholipid fatty acid composition of the studied groups.

Parameter	AN (*n* = 39)	CON (*n* = 35)
14:0 ^1^	0.24 (0.20–0.40) ^2^	0.26 (0.24–0.37)
16:0	29.53 (27.31–30.92) ^3^*	26.81 (25.55–29.05)
16:1*n*-9	0.16 (0.13–0.18)	0.17 (0.15–0.21)
16:1*n*-7	0.72 (0.52–0.95)	0.63 (0.57–0.70)
18:0	12.95 (11.52–13.68)	13.49 (12.42–14.46)
18:1tra*n*s	0.11 (0.08–0.14)	NA
18:1*n*-9	11.31 (10.70–12.63)	10.81 (10.28–11.75)
18:1*n*-7	1.87 (1.63–2.14)	1.96 (1.71–2.35)
18:2*n*-6	23.65 (21.02–25.28) *	25.53 (24.38–26.46)
18:3*n*-6	0.08 (0.05–0.11)	0.08 (0.06–0.10)
18:3*n*-3	0.21 (0.08–0.38)	0.30 (0.08–0.34)
20:0	0.06 (0.05–0.15)	0.07 (0.06–0.16)
20:1*n*-9	0.15 (0.13–0.18)	0.17 (0.14–0.19)
20:2*n*-6	0.44 (0.34–0.76)	0.40 (0.33–0.46)
20:3*n*-6	3.11 (2.35–3.61)	3.11 (2.74–3.29)
20:4*n*-6	10.46 (9.00–11.33)	10.23 (9.38–11.59)
20:5*n*-3	0.60 (0.51–0.80)	0.66 (0.54–0.81)
22:4*n*-6	0.37 (0.32–0.42)	0.37 (0.35–0.40)
22:5*n*-6	0.28 (0.20–0.35)	0.24 (0.21–0.28)
22:5*n*-3	0.92 (0.69–1.14)	0.90 (0.82–1.06)
22:6*n*-3	2.61 (1.95–3.43)	2.91 (2.17–3.80)

^1^ shorthand notation of fatty acids: number of carbon atoms; number of double bonds; *n*—number of carbon atoms from the methyl end to the nearest double bond; NA—not available; ^2^ numbers are given as median (25th–75th percentile; mol%); ^3^ statistical analysis: Mann–Whitney test: * *p* < 0.05, after adjustment for multiple comparisons using Holm’s corrections.

**Table 3:** Plasma phospholipid fatty acids of the studied groups (calculated parameters).

Parameter	AN (*n* = 39)	CON (*n* = 35)
Σ SFA	42.07 (41.18–43.57) ^2^	40.73 (39.86–43.35)
Σ MFA	14.38 (13.42–15.74)	13.82 (12.99–14.85)
Σ PUFA *n*-6	38.36 (36.97–39.77) ^3^**	39.82 (38.77–41.47)
Σ PUFA *n*-3	4.88 (3.61–5.66)	5.12 (3.65–5.82)
D5D*n*-6 (20:4*n*-6/20:3*n*-6) ^1^	3.216 (2.642–4.817)	3.338 (3.027–4.127)
D6D*n*-6 (18:3*n*-6/18:2*n*-6)	0.003 (0.002–0.005)	0.003 (0.002–0.004)
D9D16 (16:1*n*-7/16:0)	0.025 (0.019–0.032)	0.023 (0.020–0.026)
D9D18 (18:1*n*-9/18:0)	0.908 (0.829–1.031) *	0.811 (0.736–0.888)
ADA *n*-6 ^4^	0.615 (0.536–0.540)	0.572 (0.511–0.614)
22:4*n*-6/22:5*n*-6	1.369 (1.211–1.637) ^5^	1.522 (1.414–1.644)
*n*-6 EFAD index	0.030 (0.021–0.075)	0.025 (0.019–0.028)
*n*-6 EFAD index (≥0.086/<0.086)	none/39	none/35

^1^ shorthand notation of fatty acids: number of carbon atoms; number of double bonds; *n*—number of carbon atoms from the methyl end to the nearest double bond; ^2^ numbers are given as median (25th–75th percentile; mol%); ^3^ statistical analysis: Mann–Whitney test: * *p* < 0.05, ** *p* < 0.01, after adjustment for multiple comparisons using Holm’s corrections; ^4^ ADA *n*-6 (aggregated desaturase activity for PUFA *n-*6)—ratio of all PUFA *n*-6 except LA (18:2*n*-6) to LA (18:2*n*-6); ^5^ ratio 22:4*n*-6/22/5*n*-6 (adrenic to Osbond acid)—biochemical marker of functional DHA status; Abbreviations: Σsatur—sum of saturated fatty acids; ΣMFA—sum of monounsaturated fatty acids; Σ*n*-6—sum of *n*-6 polyunsaturated fatty acids; Σ*n*-3—sum of *n*-3 polyunsaturated fatty acids; *n*-6 EFAD—*n*-6 essential fatty acid deficiency; EFAD index = 16:1*n*-7/18:2*n*-6.

**Table 4:** Independent predictors of plasma HDL-cholesterol concentrations in the studied groups.

HDL-C									
**AN**					**CON**				
Model 1: FAs + BMI + FMI					Model 1: FAs + BMI + FMI				
**variable**	**estimate**	SE	*p*	Adjusted R^2^	variable	estimate	SE	*p*	Adjusted R^2^
22:5*n*-6	−0.4932	0.1321	0.0007	0.43	20:0	0.3894	0.1603	0.0208	0.15
FMI	−0.4044	0.1319	0.0042						
16:0	0.3646	0.1347	0.0106						
20:3*n*-6	−0.2568	0.1309	NS						
Model 2: FAs + Glu + Ins					Model 2: FAs + Glu + Ins				
variable	estimate	SE	*p*	Adjusted R^2^	variable	estimate	SE	*p*	Adjusted R^2^
22:5*n*-6	−0.3896	0.1514	0.0142	0.15	20:0	0.3894	0.1603	0.0208	0.15

FAs—fatty acids (see Table 3); BMI—body mass index; FMI—fat mass index; AN—anorexia nervosa; CON—control group; R^2^—coefficient of determination (in%); SE—standard error. Model 1 suggests an independent association between individual FA, BMI, FMI, and HDL-C. Model 2 suggests an independent association between individual FA, glucose, insulin, and HDL-C.

## Data Availability

The raw data supporting the conclusions of this article will be made available by the authors on request.

## Supplementary Materials

The following supporting information can be downloaded at https://www.mdpi.com/article/10.3390/nu17142347/s1, Table S1: Somatic complications of anorexia nervosa; Table S2: Plasma bile acid composition of the studied groups; Table S3: Plasma bile acid relative concentrations of the studied groups; Table S4: Independent predictors of plasma lipids (lipoproteins, respectively) in the studied groups; Table S5: Dietary and additional clinical parameters of the studied groups.

## Abbreviations

The following abbreviations are used in this manuscript:

AN-R	Anorexia nervosa (restrictive type)
FA	Fatty acid
TC	Total cholesterol
LDL-C	Low-density lipoprotein-cholesterol
HDL-C	High-density lipoprotein-cholesterol
TAG	Triacylglycerols
EDs	Eating disorders
SFA	Saturated fatty acids
MFA	Monounsaturated fatty acids
PUFA	Polyunsaturated fatty acids
SCFA	Short-chain fatty acids
CNS	Central nervous system
BA	Bile acid
DCA	Deoxycholic acid
LCA	Lithocholic acid
UDCA	Ursodeoxycholic acid
CA	Cholic acid
CDCA	Chenodeoxycholic acid
CON	Control group
REE	Resting energy expenditure
NEFA	Non-esterified fatty acids
HOMA-IR	Homeostasis model assessment method of insulin resistance
EFAD	Essential fatty acid deficiency
BMI	Body mass index
FMI	Fat mass index
D9D	Delta-9 desaturase
TUDCA	Tauroursodeoxycholic acid
apo B	Apolipoprotein B-100
DPA-6	Docosapentaenoic acid (*n*-6 family), Osbond acid
DHGLA	Dihomo-γ-linolenic acid
PA	Palmitic acid
LA	Linoleic acid
ALA	α-linolenic acid
EPA	Eisocapentaenoic acid
POA	Palmitoleic acid
LC-PUFA	Long-chain polyusaturated fatty acids
DHA	Docosahexaenoic acid
AdA	Adrenic acid
AA	Arachidonic acid
GLA	γ-linolenic acid
VLCSFA	Very-long-chain saturated fatty acids
