# Supplementary material for: Dyslipidemia in Anorexia Nervosa Is Associated with Decreased Plasma Tauroursodeoxycholic Acid and a Specific Fatty Acid Pattern
Source: Nutrients. 2025 Jul 17;17(14):2347. doi: 10.3390/nu17142347 (PMC12300223; doi:10.3390/nu17142347)
Supplement: Supplementary file 1 [file nutrients-17-02347-s001.zip › nutrients-3724420-supplementary.pdf]

nutrients

MDPI

Supplementary Tables

Dyslipidemia in Anorexia Nervosa is Associated with Decreased Plasma Tauroursodeoxycholic Acid and a Specific Fatty Acid Pattern

Supplementary Table S1. Somatic complications of anorexia nervosa.

Organ (system, respectively)	Complications
Cardiovascular	sinus bradycardia, low QRS voltage, prolonged QTc interval, arterial hypotension, orthostatic hypotension, mitral valve prolapse, decreased myocardial contractility
Dermatologic	brittle hair, hair loss, hypercarotenemia, lanugo
Oropharyngeal	parotic hyperplasia, cheilosis, teeth erosion, gingival inflammation, dysphagia
Gastrointestinal	esophageal dysmotility, nutritional hepatitis, anorectal dysfunction, gastroesophageal reflux, prolonged gut transit time, anorectal prolapses, postprandial fullness, delayed gastric emptying, gut dysbiosis, constipation, superior mesenteric artery syndrome (persistent nausea and vomiting)
Metabolic bone disease	osteopenia, osteoporosis, arrested growth
Skeletal muscles	sarcopenia
Reproductive	primary (secondary, respectively) amenorrhea, pubertal delay
Neurologic	cognitive changes, cortical atrophy with ventricular enlargement, neuropathy (sensory, motoric and visceral)
Hematologic	bone marrow suppression (with anemia, leukopenia, thrombocytopenia)
Endocrine disturbances	low testosterone and estrogen levels lower triiodothyronine and thyroxine, elevated reverse triiodothyronine, increased cortisol, higher concentration of peptide PYY and ghrelin, lower levels of leptin
Metabolic dysfunction	elevated amino acid levels, electrolyte (hyponatremia, hypokalemia), and acid-base imbalance, hypoglycemia.

Table modified from {2,3,8,12,40]

 https://doi.org/10.3390/nu17142347

Supplementary Table S2. Plasma bile acid concentrations (µmol/L) of the studied groups.

Parameter	AN (n = 21)	CON (n = 13)
LCA	0.009 (0.005 – 0.015) [1]	0.011 (0.007 – 0.020)
UDCA	0.032 (0.023 – 0.043)	0.026 (0.013 – 0.071)
HDCA	0.076 (0.068 – 0.082)	0.051 (0.029 – 0.068)
CDCA	0.161 (0.098 – 0.259)	0.196 (0.091 – 1.356)
DCA	0.323 (0.226 – 0.412)	0.319 (0.152 – 0.561)
CA	0.146 (0.055 – 0.284)	0.055 (0.018 – 0.481)
GLCA	0.014 (0.006 – 0.027)	0.021 (0.010 – 0.031)
GUDCA	0.128 (0.044 – 0.221)	0.139 (0.092 – 0.363)
GCDCA	0.383 (0.196 – 0.616)	0.764 (0.275 – 2.023)
GDCA	0.159 (0.103 – 0.296)	0.153 (0.086 – 0.375)
LCAS	0.006 (0.001 – 0.009)	0.001 (0.000 – 0.002)
GCA	0.121 (0.092 – 0.217)	0.267 (0.101 – 0.364)
TLCA	0.000 (0.000 – 0.001)	0.000 (0.000 – 0.001)
TUDCA	0.003 (0.002 – 0.006) *[2]	0.010 (0.006 – 0.021)
TCDCA	0.063 (0.036 – 0.086)	0.116 (0.071 – 0.279)
TDCA	0.038 (0.020 – 0.050)	0.025 (0.012 – 0.045)
TCA	0.036 (0.031 – 0.079)	0.079 (0.043 – 0.083)
TLCAS	0.096 (0.034 – 0.212)	0.024 (0.011 – 0.140)
Σ BA	2.677 (1.368 – 5.442)	1.903 (1.573 – 2.347)
Prim BA	0.277 (0.105 – 1.879)	0.342 (0.175 – 0.687)
Sec BA	0.365 (0.155 – 0.729)	0.349 (0.236 – 0.437)
Ter BA	0.026 (0.013 – 0.071)	0.032 (0.023 – 0.043)
Free BA	0.712 (0.316 – 2.384)	0.612 (0.500 – 0.940)
Glyco BA	1.302 (0.543 – 3.793)	0.898 (0.514 – 1.463)
Tauro BA	0.262 (0.137 – 0.455)	0.158 (0.114 – 0.209)
Sulpho BA	0.026 (0.011 – 0.158)	0.125 (0.028 – 0.212)

[1]Data are given as median (25th -75th percentile); statistical analysis: [2] Mann-Whitney test: * $p < 0.05$, after adjustment for multiple comparisons using Holm´s corrections. Abbreviations: CA - cholic acid; CDCA - chenodeoxycholic acid; GCA - glycocholic acid; DCA - deoxycholic acid; TUDCA - tauroursodeoxycholic acid; LCA - lithocholic acid; UDCA – ursodeoxycholic acid; TDCA - taurodeoxycholic acid; GDCA - glycodeoxycholic acid; GLCA - glycolithocholic acid; GUDCA - glycoursodeoxycholic acid; HCA - hyocholic acid; HDCA – hyodeoxycholic acid; TCA - taurocholic acid; UDCA - ursodeoxycholic acid; GCDCA - glycochenodeoxycholic acid; TCDCA - taurochenodeoxycholic acid; LCAS - lithocholic acid sulphate; TLCA - taurolithocholic acid; TLCAS - taurolithocholic acid sulphate; Σ BA - the sum of all BA; Prim BA - primary BA; Sec BA - secondary BA; Ter BA- tertiary BA; Free BA - free (unconjugated) BA; Glyco- (Tauro-, and Sulpho-, respectively) conjugates of BA

Supplementary Table S3. Plasma bile acid relative concentrations of the studied groups.

Parameter	AN (n = 21)	CON (n = 13)
LCA	0.442 (0.254 – 0.635)[1]	0.303 (0.151 – 0.853)
UDCA	1.163 (0.921 – 1.766)	1.599 (0.495 – 1.943)
HDCA	2.569 (2.090 – 3.172)	1.143 (1.053 – 2.645)
CDCA	6.621 (3.741 – 13.359)	10.536 (5.492 – 21.340)
DCA	15.009 (12.949 – 18.914)	9.373 (6.604 – 11.650)
CA	5.779 (3.497 – 14.950)	2.047 (1.232 – 8.231)
GLCA	0.511 (0.291 – 1.342)	0.597 (0.457 – 0.948)
GUDCA	5.493 (2.295 – 11.652)	6.259 (4.913 – 9.036)
GCDCA	16.862 (12.024 – 22.917)	29.724 (23.123 – 32.136)
GDCA	7.960 (6.139 – 13.034)	6.146 (3.258 – 8.835)
LCAS	0.161 (0.058 – 0.548)	0.041 (0.015 – 0.102)
GCA	6.702 (4.797 – 9.112)	6.993 (5.476 – 11.665)
TLCA	0.012 (0.006 – 0.036)	0.014 (0.010 – 0.029)
TUDCA	0.140 (0.068 – 0.251) *[2]	0.397 (0.295 – 0.493)
TCDCA	3.253 (1.620 – 4.244)	4.167 (3.482 – 5.291)
TDCA	1.952 (1.105 – 2.580)	0.936 (0.539 – 1.252)
TCA	2.005 (1.489 – 4.014)	2.374 (1.591 – 3.039)
TLCAS	5.505 (1.550 – 12.466)	1.815 (0.893 – 2.197)
Prim BA	17.751 (7.983 – 34.055)	12.102 (7.366 – 29.571)
Sec BA	17.195 (14.091 – 19.483)	10.451 (7.873 – 15.787)
Ter BA	1.163 (0.921 – 1.766)	1.599 (0.495 – 1.943)
Free BA	35.907 (21.921 – 52.049)	37.387 (22.557 – 47.358)
Glyco BA	47.845 (29.277 – 68.772)	53.441 (40.525 – 61.883)
Tauro BA	8.527 (4.630 – 9.986)	8.065 (6.212 – 10.064)
Sulpho BA	5.730 (1.736 – 11.441)	1.917 (0.905 – 2.926)

[1]Data are given as median (25th -75th percentile); statistical analysis: [2] Mann-Whitney test: * $p < 0.05$, after adjustment for multiple comparisons using Holm´s corrections. Abbreviations: CA - cholic acid; CDCA - chenodeoxycholic acid; GCA - glycocholic acid; DCA - deoxycholic acid; TUDCA - tauroursodeoxycholic acid; LCA - lithocholic acid; UDCA – ursodeoxycholic acid; TDCA - taurodeoxycholic acid; GDCA - glycodeoxycholic acid; GLCA - glycolithocholic acid; GUDCA - glycoursodeoxycholic acid; HCA - hyocholic acid; HDCA – hyodeoxycholic acid; TCA - taurocholic acid; UDCA - ursodeoxycholic acid; GCDCA - glycochenodeoxycholic acid; TCDCA - taurochenodeoxycholic acid; LCAS - lithocholic acid sulphate; TLCA - taurolithocholic acid; TLCAS - taurolithocholic acid sulphate; Σ BA - the sum of all BA; Prim BA - primary BA; Sec BA - secondary BA; Ter - tertiary BA; Free BA - free (unconjugated) BA; Glyco- (Tauro-, and Sulpho-, respectively) conjugates of BA

Supplementary Table S4. Independent predictors of plasma lipids (lipoproteins, respectively) in the studied groups.

TAG

AN					CON				
**Model 1: FAs+BMI+FMI**					**Model 1: FAs+BMI+FMI**				
**variable**	**estimate**	**SE**	**p**	**Adjusted R^2**	**variable**	**estimate**	**SE**	**p**	**Adjusted R^2**
20:0	-0.4580	0.1330	0.0015	0.39	22:5n-6	-0.6072	0.1453	0.0002	0.37
16:1n-9	0.3414	0.1330	0.0146		20:5n-3	0.3211	0.1453	0.0344	
**Model 2: FAs+Glu+Ins**					**Model 2: FAs+Glu+Ins**				
**variable**	**estimate**	**SE**	**p**	**Adjusted R^2**	**variable**	**estimate**	**SE**	**p**	**Adjusted R^2**
20:0	-0.5122	0.1299	0.0004	0.45	22:5n-6	-0.6072	0.1453	0.0002	0.37
16:1n-9	0.3481	0.1273	0.0097		20:5n-3	0.3211	0.1453	0.0344	
Insulin	-0.2662	0.1278	0.0446						

LDL-C/HDL-C

AN					CON				
**Model 1: FAs+BMI+FMI**					**Model 1: FAs+BMI+FMI**				
**variable**	**estimate**	**SE**	**p**	**Adjusted R^2**	**variable**	**estimate**	**SE**	**p**	**Adjusted R^2**
22:4n-6	0.5366	0.1387	0.0004	0.29	16:0	-0.6739	0.1286	0.0001	0.45
**Model 2: FAs+Glu+Ins**					**Model 2: FAs+Glu+Ins**				
**variable**	**estimate**	**SE**	**p**	**Adjusted R^2**	**variable**	**estimate**	**SE**	**p**	**Adjusted R^2**
22:4n-6	0.5366	0.1387	0.0004	0.29	16:0	-0.6739	0.1286	0.0001	0.45

TC

AN					CON				
**Model 1: FAs+BMI+FMI**					**Model 1: FAs+BMI+FMI**				
**variable**	**estimate**	**SE**	**p**	**Adjusted R^2**	**variable**	**estimate**	**SE**	**p**	**Adjusted R^2**
22:4n-6*	0.5856	0.1587	0.0007	0.28	16:0	-0.7924	0.1529	0.0001	0.46
18:1n-9	-0.3598	0.1587	0.0295		Σn-6	-0.3724	0.1529	0.0206	
**Model 2: FAs+Glu+Ins**					**Model 2: FAs+Glu+Ins**				
**variable**	**estimate**	**SE**	**p**	**Adjusted R^2**	**variable**	**estimate**	**SE**	**p**	**Adjusted R^2**
22:4n-6	0.5856	0.1587	0.0007	0.28	16:0	-0.4767	0.1299	0.0009	0.51
18:1n-9	-0.3598	0.1587	0.0295		Glucose	-0.4068	0.1299	0.0037	

LDL-C

AN					CON				
**Model 1: FAs+BMI+FMI**					**Model 1: FAs+BMI+FMI**				
**variable**	**estimate**	**SE**	**p**	**Adjusted R^2**	**variable**	**estimate**	**SE**	**p**	**Adjusted R^2**
22:4n-6	0.6432	0.1528	0.0002	0.33	16:0	-0.9173	0.1247	0.0001	0.64
18:1n-9	-0.3654	0.1528	0.0222		Σn-6	-0.3099	0.1247	0.0183	
**Model 2: FAs+Glu+Ins**					**Model 2: FAs+Glu+Ins**				
**variable**	**estimate**	**SE**	**p**	**Adjusted R^2**	**variable**	**estimate**	**SE**	**p**	**Adjusted R^2**
22:4n-6	0.6432	0.1528	0.0002	0.33	16:0	-0.6649	0.1089	0.0001	0.65
18:1n-9	-0.3654	0.1528	0.0222		Glucose	-0.3038	0.1089	0.0088	

apoB

AN					CON				
**Model 1: FAs+BMI+FMI**					**Model 1: FAs+BMI+FMI**				
**variable**	**estimate**	**SE**	**p**	**Adjusted R^2**	**variable**	**estimate**	**SE**	**p**	**Adjusted R^2**
ADA n-6	0.4261	0.1517	0.0080	0.21	16:0	-0.9775	0.1826	0.0001	0.61

ΣMFA	-0.3046	0.1517	NS		18:2n-6	-0.8801	0.1722	0.0001	
					20:4n-6	-0.5257	0.1555	0.0021	
					20:0	0.4421	0.1597	0.0097	
					BMI	-0.3081	0.1225	0.0177	
**Model 2: FAs+Glu+Ins**					**Model 2: FAs+Glu+Ins**				
**variable**	**estimate**	**SE**	**p**	**Adjusted R^2**	**variable**	**estimate**	**SE**	**p**	**Adjusted R^2**
ADA n-6	0.4063	0.1486	0.0096	0.22	Ins	-0.4192	0.1186	0.0014	0.61
Insulin	-0.3166	0.1486	0.0400		18:0	-0.9613	0.5246	NS	
					18:1n-9	1.8448	0.5896	0.0040	
					D9D18	-2.3661	0.8646	0.0105	
					D6Dn-6	0.2519	0.1245	NS	

**Non-HDL-C**									
**AN**					**CON**				
**Model 1: FAs+BMI+FMI**					**Model 1: FAs+BMI+FMI**				
**variable**	**estimate**	**SE**	**p**	**Adjusted R^2**	**variable**	**estimate**	**SE**	**p**	**Adjusted R^2**
22:4n-6	0.6310	0.1547	0.0002	0.32	16:0	-0.8705	0.1378	0.0001	0.56
18:1n-9	-0.3160	0.1547	0.0485		Σn-6	-0.3560	0.1378	0.0145	
**Model 2: FAs+Glu+Ins**					**Model 2: FAs+Glu+Ins**				
**variable**	**estimate**	**SE**	**p**	**Adjusted R^2**	**variable**	**estimate**	**SE**	**p**	**Adjusted R^2**
22:4n-6	0.6310	0.1547	0.0002	0.32	16:0	-0.8705	0.1378	0.0001	0.56
18:1n-9	-0.3160	0.1547	0.0485		Σn-6	-0.3560	0.1378	0.0145	

* FAs – fatty acids (see Table 3); BMI – body mass index; FMI – fat mass index; AN – anorexia nervosa; CON – control group; R^2 – coefficient of determination (in %); SE – standard error

Model 1 suggests an independent association between individual FAs, BMI, FMI, and plasma lipids (lipoproteins, respectively)

Model 2 suggests an independent association between individual FAs, glucose, and insulin

Supplementary Table S5: Dietary and additional clinical parameters of the studied groups

Parameter	Anorexia nervosa (n=39)	Healthy controls (n=35)
BW loss (% of BW/ 6 months before diagnosis)	21.3 ± 9.9*	3.5 ± 2.1[a]
SBP (mm Hg)	98 ± 12	100 ± 23
DBP (mm Hg)	65 ± 8	61 ± 15
Energy Intake (kcal/kg BW/day)	22.7 ± 11.1**	31.8 ± 6.3
Fat intake (g/kg BW/day)	0.7 ± 0.4**	1.1 ± 0.4
Cholesterol intake (mg/kg BW/day)	3.1 ± 1.7*	4.3 ± 1.0
Protein intake (g/kg BW/day)	1.0 ± 0.6	1.3 ± 0.6
Fiber intake (g/kg BW/day)	0.3 ± 0.2	0.2 ± 0.2
Saturated fatty acid intake (g/kg BW/day)	0.2 ± 0.1*	0.3 ± 0.2
Diagnosis delay (months)	9.8 ± 8.5	NA/none
Duration of primary or secondary amenorrhea (months)	7.5 ± 5.5	none

Abbreviations used: BW – body weight; SBP – systolic blood pressure, DBP – diastolic blood pressure

[a]/ Data are given as mean ± S.D; statistical analysis: unpaired Student´s t-test with Holm correction for multiple comparisons comparing AN patient group and healthy controls: * $p < 0.05$; ** $p < 0.01$.
